# Characterization, Stability, and Antibrowning Effects of Oxyresveratrol Cyclodextrin Complexes Combined Use of Hydroxypropyl Methylcellulose

**DOI:** 10.3390/foods11162471

**Published:** 2022-08-16

**Authors:** Jianfei He, Huai-Yu Chen, Hongbin Chen, Baobei Wang, Fengxian Guo, Zong-Ping Zheng

**Affiliations:** 1 Fujian Province Key Laboratory for the Development of Bioactive Material from Marine Alge, College of Oceanology and Food Science, Quanzhou Normal University, Quanzhou 362000, China; 2 Department of Biomedical Science, University of Copenhagen, 2200 Copenhagen, Denmark

**Keywords:** oxyresveratrol (Oxy), cyclodextrin (CD) inclusion complex, hydroxypropyl methylcellulose (HPMC), storage stability, interaction, antibrowning activity

## Abstract

Oxyresveratrol (Oxy) has attracted much attention by employing it as an antibrowning agent in fruits and vegetables. In this study, the formation of cyclodextrin (CD) inclusion exhibited a certain protective effect on Oxy oxidative degradation, while hydroxypropyl-β-cyclodextrin (HP–β-CD) inclusion complex showed stronger stabilizing effects than those of β-cyclodextrin (β-CD). The combined use of CD and hydroxypropyl methylcellulose (HPMC) greatly improved the stability of Oxy–CD inclusion complexes, with approximately 70% of the *trans*-Oxy retained after 30 days of storage under light conditions at 25 °C. The results of the interaction between CD and Oxy determined by phase solubility studies and fluorescence spectroscopic analysis showed that the binding strength of CD and Oxy increased in the presence of HPMC. Moreover, Oxy combined with ascorbic acid and HPMC showed an excellent antibrowning effect on fresh-cut apple slices during the 48 h test period, indicating that adding HPMC as the third component will not influence the antibrowning activity of Oxy.

## 1. Introduction

Oxyresveratrol (2,3′,4,5′-tetrahydroxystilbene, Oxy), also known as oxystilbene triphenyl, is a kind of plant antitoxin [1] that exhibits multiple biological activities including neuroprotection [2], antibacterial [3], antiviral [4], anticancer [5], anti-aging [6], and antioxidant activity [7]. It is a 2′-hydroxylated derivative of resveratrol; however, it has less cytotoxicity, better solubility, higher cell permeability, and greater bioavailability than resveratrol [8,9]. Greater free-radical scavenging ability than ascorbic acid (VC) is found in Oxy, and there is much evidence to support the role of other antioxidants in synergistically enhancing the antioxidant effects of Oxy [10,11]. Therefore, Oxy is promising in functional food and medicine ingredients [12], especially to be used as an antibrowning additive to maintain the freshness of fruits and vegetables due to its powerful tyrosinase inhibitory effect [13].

Similar to resveratrol, Oxy is thought to be an extremely photosensitive compound that is easily affected by the external environment, including light, temperature, pH, and oxidants, and undergoes oxidative degradation or isomerization [14]. Oxy mainly exists in the *trans*-form, and other structural forms, such as *cis*-form and glucoside isomers, have also been found. The physiological activities of Oxy glycosides are generally believed weaker than Oxy, while the *cis*-structure generally exhibits lower biological activities than *trans*-forms [15,16]. These two isomeric forms can be transformed into each other. Especially under ultraviolet radiation, *trans*-form Oxy is easily isomerized into the *cis* structure [17]. Thus, Oxy usually needs to be stored in dark conditions.

If this effective antioxidant compound is to be used successfully in both food and pharmaceutical industries as an ingredient of nutraceuticals, it is desirable not only to improve its water solubility but also to stabilize Oxy, considering its poor bioavailability [18]. Many carriers have been used previously to improve the Oxy bioavailability in solution, including cell encapsulation technology [19], lactoglobulin binding [20], microemulsions [21], cyclodextrins (CDs) [22], liposomes [23], and water-soluble polysaccharides [24], as well as solid lipid nanoparticles (SLNs) and nano lipid carriers (NLCs) [25,26]. Among them, the CD is the most common encapsulation material to enhance the solubility of hydrophobic molecules. These torus-shaped oligosaccharides are made up of α-(1,4) linked glucose units, with high solubility in an aqueous solution, and can wrap hydrophobic molecules in its cavity [27]. The strategy of using β-cyclodextrin (β-CD) or hydroxypropyl-β-CD (HP–β-CD) to improve the solubility of Oxy has been described in our previous work, together with characterizing the solid states of this binary system [28]. However, it must be noted that the problem of poor stability of the Oxy–CD complex still exists [29]. It has been proved that the addition of hydroxypropyl methylcellulose (HPMC) helps to enhance the storage stability [30] and the activity of some lipophilic molecules, such as resveratrol and piperine [31,32]. There are also some reports that HPMC can be used as a mediator to enhance the complexation efficiency of CD to guest molecules [33,34]. Compared with the binary system, the formed guest-molecule–CD–polymer ternary system may help to achieve better physical and chemical properties of the guest molecule, such as antioxidant and antibacterial activities [31,32]. Although the exact nature of the polymer:CD interaction, as well as the specific effects of complexation on molecular stability and activity, is still not known.

In our literature survey, there were also no published reports on the ternary inclusion of Oxy with CD–polymers. Based on the previous reports on the role of HPMC in the ternary system [33,34], it is speculated that the presence of HPMC will further enhance the complexing ability of CD and Oxy, thereby improving the stability of Oxy in solution, and will not adversely affect the antibrowning activity of Oxy. To investigate the effect of HPMC on the Oxy–CD binary system and to evaluate the possible effect of complexation on the stability and antibrowning activity of Oxy, we have therefore interrogated the features of Oxy–CD, Oxy–HPMC, and Oxy–CD–HPMC solutions, and present data which indicate that using HPMC as the third component to improve the stability of the Oxy and β-CD or HP–β-CD inclusion complex (Oxy–β-CD and Oxy–HP–β-CD) in solution without influence its antibrowning activity to fresh-cut apple slices.

## 2. Materials and Methods

### 2.1. Chemicals and Materials

The 2-hydroxypropyl-β-cyclodextrin (HP–β-CD, ≥98% pure) and β-cyclodextrin (β-CD, ≥98% pure) were purchased from Jiangsu Fengyuan Biotechnology Co., Ltd. (Suqian, China). Oxyresveratrol (Oxy, ≥98% pure) was purchased from Hangzhou Great Forest Biomedical Ltd. (Hangzhou, China). Ascorbic acid (VC) was purchased from Sigma Chemical Co. (St. Louis, MO, USA). HPLC grade formic acid, chitosan ≥75% (deacetylated), carboxymethyl cellulose (CMC) of medium viscosity, and hydroxypropylmethylcellulose (HPMC) of 4000 CP were purchased from Shanghai Aladdin Chemical Reagent Co., Ltd. (Shanghai, China). HPLC grade methanol was purchased from J&K Scientific (Beijing, China). High-performance liquid chromatography (HPLC)-grade formic acid was purchased from Shanghai Aladdin Chemical Reagent Co., Ltd. (Shanghai, China). Apples (cultivar: Fuji) with uniform size (300 ± 20 g) and color (commercial maturity) were purchased from the local supermarket and stored at 4 °C overnight.

### 2.2. Solubilization Effect of Water-Soluble Polysaccharides on Oxy

Due to their nontoxic and biodegradable properties, many water-soluble polysaccharides have been widely used in both the food and pharmaceutical industries. The chitosan and cellulose derivatives (CMC and HPMC) selected in this study are typical polysaccharides that have been extensively studied, and there are many research examples for improving the solubility, stability, and bioavailability of active ingredients [35]. Combined with previous findings that the effect of the water-soluble polysaccharide operates in a dose-dependent manner. The presence of a small amount of polymer in the solution can significantly increase the complexing capacity of CD and improve the availability of insoluble components in CD aqueous solutions [36]; therefore, polymer concentrations not exceeding 5 mg/mL were investigated in the following experiments. First, 5 mg Oxy was added to 5 mL chitosan, CMC, and HPMC solution with concentrations varying from 0 to 5 mg/mL, respectively. The mixture was stirred by a Genius 3 vortex and RW 20D magnetic stirrer (IKA laboratory technology, Staufen, Germany) for 48 h at 20 °C. After equilibration, the mixed solution was centrifuged at 8000 rpm for 20 min. The supernatant was diluted with methanol to the required concentration (5 times volume) and then filtered by a 0.2 μm membrane filter. HPLC was used to determine the Oxy concentration subsequently, the HPLC analysis method referred to our previous study (provided in the detail in Figures S1 and S2) [28]. Each sample was replicated three times.

### 2.3. Phase Solubility Study

Here, 20 mg Oxy was added to 5 mL β-CD or HP–β-CD solutions (0–10 mM) in the presence or absence of HPMC (1 mg/mL) [28]; the mixture was stirred by a Genius 3 vortex and RW 20D magnetic for 48 h at 20 °C. After equilibrium, the mixed solution was centrifuged at 8000 rpm for 20 min to get rid of the precipitate. The supernatant was diluted with methanol (10 times volume) and then analyzed by HPLC at 325 nm to determine the concentration of Oxy in an aqueous solution (provided in the detail in Figures S1 and S2). The phase solubility diagram was then plotted with CD concentration as abscissa and drug concentration as ordinate.

### 2.4. Preparation of Oxy–HPMC, Oxy–CD, and Oxy–CD–HPMC Solutions

Oxy–CD inclusion complexes were prepared according to the method we established before [28]. β-CD or HP–β-CD was dissolved in water (10 mM), and then Oxy was added in batch at the 1:1 (β-CD: Oxy) or 1:1.3 (HP–β-CD: Oxy) ratio with vigorous stirring for 24 h until equilibrium. After that, the solution was filtered with a 0.2 μm membrane filter and spray-dried with Mini Spray Dryer B-290 (Büchi, Flawil, Switzerland). The drying conditions were as follows: flow rate, 3.5 mL/min; inlet temperature, 160 °C; outlet temperature, 80 °C; and air-flow rate, 300 NI/h. Oxy–CD powders were stored in light-proof sealed bottles. The Oxy content of the obtained inclusion compound was 207.19 mg/g βCD inclusion compound and 198.30 mg/g HP–β-CD inclusion compound, respectively. Oxy and Oxy–CD solutions (0.1 mM) were prepared by dissolving Oxy or Oxy–CD power in water under continuous stirring. Oxy–HPMC and Oxy–CD–HPMC solutions (0.1 mM) were prepared by dissolving Oxy or Oxy–CD to 1 mg/mL HPMC solution under continuous stirring. The mixture solutions were followed by stirring for 24 h until equilibrium and filtered through a 0.2 μm membrane filter before being used.

### 2.5. Fluorescence Spectroscopy Analysis

To study the interactions among Oxy–CD, Oxy, and HPMC, Oxy–CD powder was dissolved in 0–5 mg/mL HPMC solution (0.1 mM) and stirred continuously for 24 h until equilibrium. The fluorescence spectra of the solutions were recorded on a Hitachi F-2700 fluorescence spectrophotometer (Hitachi High-Technology Corporation, Tokyo, Japan). The excitation wavelength was 288 nm (10 nm slit width), and the emission spectra were monitored from 300 to 550 nm, with intervals of 0.5 nm. The experiment was repeated three times. Oxy–CD inclusion complex was prepared as described in Section 2.4.

### 2.6. Particle Size Analysis

Oxy–HPMC, Oxy–CD, and Oxy–CD–HPMC solutions were prepared as described in Section 2.4, and the mean particle size and particle size distribution of these solutions were evaluated using a Zetasizer Nano ZS photon correlation spectroscopy (Marvern Instruments; Worcestershire, UK) equipped with a 4 mW helium/neon laser employing a wavelength of 633 nm and a fixed angle of 173.

### 2.7. Stability Study of Oxy–CD and Oxy–CD–HPMC Solutions

The long-term stability of Oxy, Oxy–CD, Oxy–HPMC, and Oxy–CD–HPMC solutions (prepared as described in Section 2.4) was studied by static storage under different experimental conditions (including temperature, light, and pH) for 30 days, and the percentage of remaining Oxy (both *trans*- and *cis*-forms) was recorded by monitor Oxy content with HPLC at a certain time interval. All samples were stored in well-closed glass bottles. UV/sunlight radiation is thought to induce isomerization from *trans*- to *cis*-form, resulting in the formation of a mixture consisting of Oxy in *trans*- and *cis*-forms [37,38,39]. On the supposition that the isomerization of *trans*-Oxy by radiation is equal to the generation of *cis*-Oxy and the molar absorptivity of *cis* was the same as that of *trans*-form at the wavelength of maximum absorbance [40]. A calibration curve for the quantitation of *cis*-Oxy at 325 nm was obtained by exposing the *trans*-Oxy methanol solution to a three-purpose ultraviolet analyzer WFH-203(ZF-1) at 365 nm for different times (up to 5 h) and reanalyzing by HPLC. The results in the concentration range of 9.4–175.5 mg/L were fitted to linear regression analysis (provided in the detail in Figures S1–S3).

### 2.8. Antibrowning Effects of Oxy–CD–HPMC and Oxy–CD–HPMC + VC on Fresh-Cut Apple Slices

Fresh-cut apple slices were used as a model to investigate the antibrowning effect of Oxy in food systems. The apples were cleaned, peeled, and cut into 1–1.5 cm thick slices. Then the surface was dried with filter paper, dipped into 50 mL solutions of different treatments for 4 min, and drained. The test solutions used for the above samples are shown in Table 1. These apple slices were then put into separate petri dishes and stored for 48 h (kept at room temperature with open access to air). The chromatism of each experimental group slice was examined on a high-performance color measurement spectrophotometer (UltraScan Pro 1166, HunterLab, Beijing, China), expressed as L [brightness (0–100)], a [red (−) to green (+)] and b [blue (−) to yellow (+)] values. Total color difference (Δ*E*) was always used to evaluate the antibrowning effect of different treatments [41], which was calculated as follows [42]:

Δ*E* = [(L*_t_ − L*_initial_)^2^ + (a*_t_ − a*_initial_)^2^ + (b*_t_ − b*_initial_)^2^]^0.5^ (1)

Browning index (*BI*) is another important color parameter that indicates the amount of enzymatic and non-enzymatic browning reaction during storage [43], which was calculated as follows:

*BI* = [100(x − 0.31)]/(0.172) (2)

where x is called the chromaticity coordinate, obtained from the CIE L*a*b* coordinates according to the following formula:

x = (a*_t_ + 1.75 L*_t_)/(5.646 L*_t_ + a*_t_ − 3.012 b*_t_) (3)

Measurements were made immediately following each treatment and at timed intervals of 0, 3, 6, 9, 12, 24, and 48 h thereafter. Oxy–HPMC, Oxy–CD, and Oxy–CD–HPMC solutions were prepared as described above (Section 2.4), but the concentration of Oxy was increased to 1.6 mM. All experiments were performed six times.

### 2.9. Statistical Analysis

Statistical analyses were performed using OriginPro 2019 (version 9.6.0.172; OriginLab, Northampton, MA, USA) and Graphpad Prism (version 9; GraphPad Software, San Diego, CA, USA). The results were expressed as mean values ± standard deviation (SD). A two-way ANOVA with Tukey’s multiple comparison test was performed on data from at least three independent experiments, with *p* < 0.05 considered significant.

## 3. Results and Discussion

### 3.1. Solubility of Oxy in Various Water-Soluble Polymers

**Figure 1:**
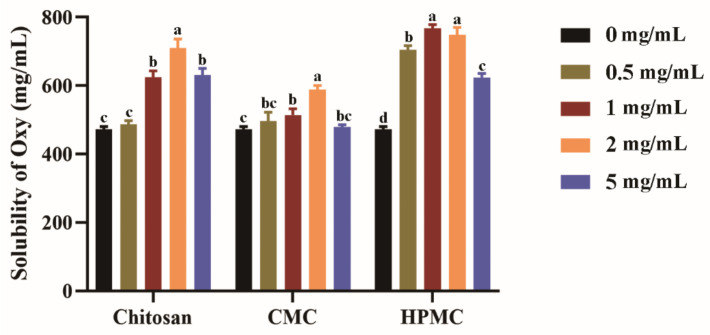
The effect of chitosan, carboxymethylcellulose (CMC), and hydroxypropyl methylcellulose (HPMC) on the solubility of Oxy in water at 20 °C (*n* = 3). Data were presented as mean ± SD, and significant differences among 0 mg/mL (no water-soluble polymer added) and different concentrations of polymer are shown with lower case letters a–d.

As previously reported, water-soluble polymers can help to improve the solubility of hydrophobic molecules [33,35], and the polymer concentration is critical for this effect [44]. Figure 1 revealed the effects of chitosan, CMC, and HPMC (0–5 mg/mL) on the solubility of Oxy in water. The presence of these three polymers showed a similar solubilization pattern, with the solubility of Oxy increasing first and then decreasing within a certain polymer concentration range. When compared to chitosan and CMC, Oxy has better solubility in the same concentration of HPMC solution. Especially at the concentration of 1 mg/mL HPMC, it displayed the best efficacy, increasing Oxy solubility from 0.47 mg/mL to 0.77 mg/mL. The addition of higher concentrations of HPMC to the solution did not result in further increases in solubility; 1 mg/mL HPMC in solution was therefore used for further studies. The improved properties exhibited by the above water-soluble polymers in increasing the solubility and the degree of supersaturation of Oxy may be the effect of the polymer gel layer formed in the solution. Polymers can form intramolecular and intermolecular hydrogen and hydrophobic bonds. In the dissolved state, these polymers rapidly hydrate and swell, forming a gel layer around the dry core of the polymer matrix. During this process, their intramolecular bonds are disrupted, so the polymers may form new hydrogen or hydrophobic interactions with hydrophobic molecules, thereby delaying their precipitation [35].

### 3.2. Study on the Interaction among Oxy–CD, and HPMC

#### 3.2.1. Phase Solubility Study

**Figure 2:**
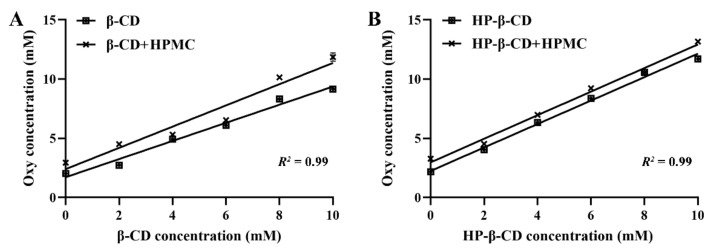
Phase solubility diagrams of Oxy and CD with or without the presence of 1 mg/mL HPMC solutions at 20 °C (*n* = 3, if no error bars are displayed, errors were smaller than symbols). (**A**) β-CD; (**B**) HP–β-CD.

The phase solubility diagram was performed to investigate the interactions between Oxy and CD with or without the presence of 1 mg/mL HPMC. As shown in Figure 2, with the increasing amount of β-CD and HP–β-CD, the solubility of Oxy observed in 1 mg/mL HPMC solution (*R*^2^ ≥ 0.99) exhibited a linear increase, demonstrating that CD can still form inclusion compound with Oxy in a stoichiometry ratio of 1:1 with the presence of HPMC [28]. In addition, HPMC can further improve the binding affinity between Oxy and these two CDs, which is manifested as higher encapsulation constants (K_F_) [28]. The K_F_ value of β-CD was increased from 1.90 × 10^4^ M^−1^ to 3.60 × 10^4^ M^−1^ (Figure 2A), while it changed from 3.59 × 10^4^ M^−1^ to 6.97 × 10^4^ M^−1^ for HP–β-CD (Figure 2B) in the absence and presence of HPMC in solution. Higher K_F_ values for ternary systems give more stable complexes than binary systems. This behavior differs from that of the molecular form of the naproxen when complexed with β-CD in the presence of PVP [45] but is in good agreement with the findings of Sultan et al. that the presence of HPMC enhances the interaction between small lipophilic molecules and CDs [32].

#### 3.2.2. Fluorescence Spectroscopy Analysis

**Figure 3:**
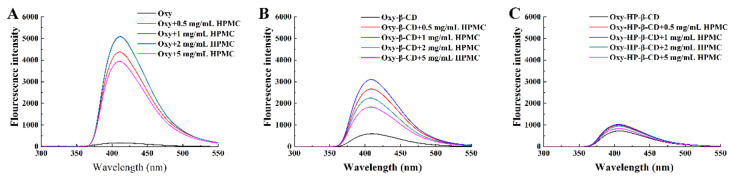
Exemplary fluorescence spectra of Oxy in the presence of various concentrations of HPMC. (**A**) Oxy solution; (**B**) Oxy–β-CD solution; (**C**) Oxy–HP–β-CD solution.

The fluorescence spectra of Oxy in the absence and presence of β-CD or HP–β-CD at different concentrations were given in our previous report [28,46]. Both β-CD and HP–β-CD exhibited significant effects on the fluorescence spectrum of Oxy, in terms of peak intensity and maximum emission wavelength. These data suggest that a stable inclusion complex is formed between Oxy and β-CD or HP–β-CD. The CD cavity provides an apolar environment for the Oxy molecule and thus increases the quantum yield of the fluorescence of Oxy [47]. To investigate the interactions among Oxy–CD, and HPMC, the fluorescence spectra of Oxy and Oxy–CD inclusion complex in the presence of various concentrations of HPMC were therefore recorded here (Figure 3). As shown in Figure 3A, the fluorescence signal of Oxy in solution was significantly enhanced in the presence of all tested HPMC concentrations, although the magnitude of the increase was slightly reduced at high HPMC concentrations. However, there was no shift in the maximum emission wavelength, indicating that Oxy may not interact with HPMC directly. The observed enhancement of the fluorescence signal may be related to the increase in solution viscosity. It has been previously reported that the collision between the fluorescent substance and Oxy decreases as the viscosity of the solution increases [48]. The reduction of this deactivation process is manifested as an enhancement in the fluorescence intensity of Oxy.

Exemplary fluorescence spectra of Oxy–β-CD and HP–β-CD inclusion complex in the presence of various concentrations of HPMC were shown in Figure 3B,C. A weak blue shift and enhancement of the fluorescence signal of Oxy can be observed in HPMC solutions. The enhancement of the fluorescence intensity suggests that the addition of HPMC helps to increase the binding affinity between Oxy and CD; the Oxy molecule encapsulated into the CD cavity is further restricted in freedom, increasing the fluorescence quantum yield of Oxy. On the other hand, the influence of HPMC on solution viscosity may also contribute to this result. Oxy–β-CD and HP–β-CD inclusion complexes showed different fluorescence intensity enhancements in the presence of HPMC, which was greater for the Oxy–β-CD inclusion compound than that of HP–β-CD. One possible reason is that Oxy in the Oxy–β-CD complex is easier to leave the CD cavity than Oxy–HP–β-CD due to the low binding activity, so it is more susceptible to HPMC [28,46]. This fact, combined with the higher complexation constants obtained from phase-solubility analysis, suggests that polymers influence the complexation of CD by forming ternary complexes or co-complexes, rather than by interfering with individual insoluble small molecules or self-aggregated CD. This observation is consistent with previous findings showing that polymer molecules act as bridges for the complexation between Oxy–CD [45].

#### 3.2.3. Particle Size Analysis

**Figure 4:**
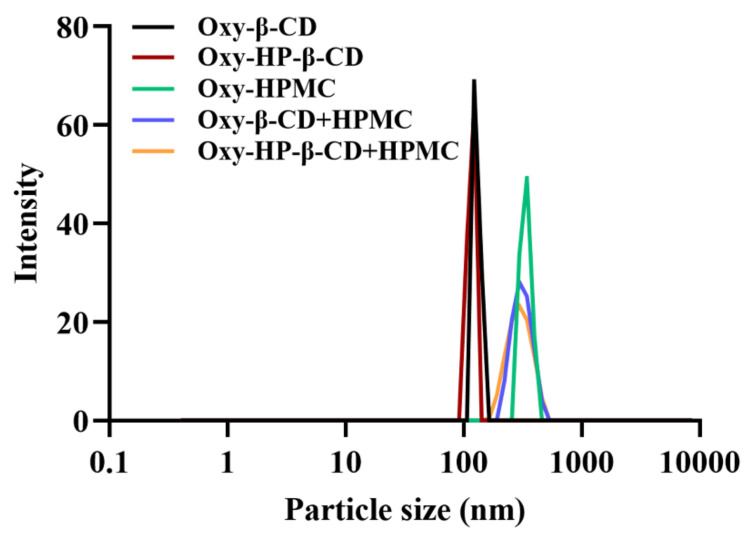
Intensity particle size distribution of Oxy–CD, Oxy–HPMC, and Oxy–CD–HPMC solution at the Oxy concentration of 1mM were measured on a Zetasizer Nano ZS.

The observed particle sizes of Oxy–HPMC, Oxy–CD, and Oxy–CD-HPMC were consistent with solution fluorescence data, indicating Oxy–CD-HPMC may form ternary complexes. As shown in Figure 4, the average particle diameters of Oxy–β-CD and HP–β-CD inclusion complexes were about 128.22 ± 6.76 nm and 116.14 ± 4.23 nm, respectively, with a narrow particle size distribution. In Oxy–HPMC solution, the average particle size was about 335.59 ± 6.34 nm. The particle size of the solution increased significantly after the formation of the ternary complex, 295.30 ± 5.76 nm and 296.60 ± 5.35 nm for Oxy–β-CD–HPMC and HP–β-CD–HPMC, respectively, and the particle size distribution was relatively uniform. Oxy–CD complexes in solution have high CD mobility, and non-ionic polymers may interact with the outer surfaces of both CD and Oxy–CD complexes, possibly enhancing the interaction strength of Oxy with CD by forming large Oxy–CD complex aggregates, thereby improving the stability of Oxy in solution [33]. This supports the idea that adding a small amount of water-soluble polymer to the aqueous solution of Oxy–CD complexes can play a stabilizing role.

### 3.3. Long-Term Storage

#### 3.3.1. Visual Appearance

**Figure 5:**
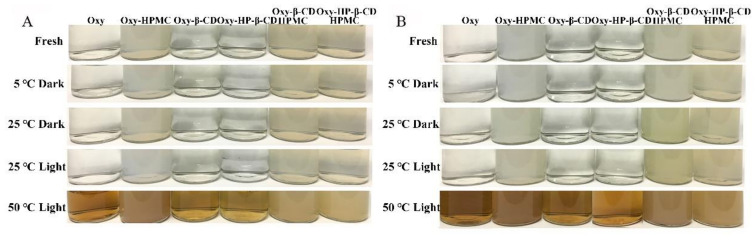
The visual appearance of Oxy, Oxy–HPMC, Oxy–CDs, and Oxy–CDs-HPMC solutions under different conditions during 30 days of storage. (**A**) Water; (**B**) acidic solution (pH 3.6).

The appearance of Oxy–HPMC, Oxy–CD, and Oxy–CD–HPMC solutions after long-term storage was characterized in terms of visual inspection, as shown in Figure 5A (in water) and Figure 5B (in acidic solution, pH 3.6). The appearance of freshly prepared Oxy and Oxy–β-CD/HP–β-CD solutions was light yellow, clear, and transparent liquid, whereas it changed to translucent and milky with the presence of 1 mg/mL HPMC. All solutions tested had good fluidity without layering (Figure 5A). After long-term storage at 4 °C and 25 °C, test solutions were still transparent and clear by visual observation; however, some white solid precipitates were found in Oxy water solution at low temperature (4 °C). The absence of Oxy precipitation in HPMC solution may be because the water-soluble polymer can help to maintain drug supersaturation by acting as crystallization inhibitors [49]. At 50 °C, the color of all tested Oxy solutions darkened clearly, which may be caused by the oxidation of Oxy [21]. The addition of HPMC does not seem to exhibit an obvious color protection effect. Oxy and Oxy–HPMC solutions changed from light yellow to deep yellow, whereas Oxy–CD and Oxy–CD-HPMC solutions changed to yellow. Nevertheless, the color change of the ternary composite solution was minimal in comparison. Under acidic conditions (Figure 5B), the visual color change of each sample displayed the same tendency as that in water, whereas the transparency of the HPMC solutions was reduced and the color became darker after high-temperature storage.

#### 3.3.2. The Stability of Oxy in Water

**Figure 6:**
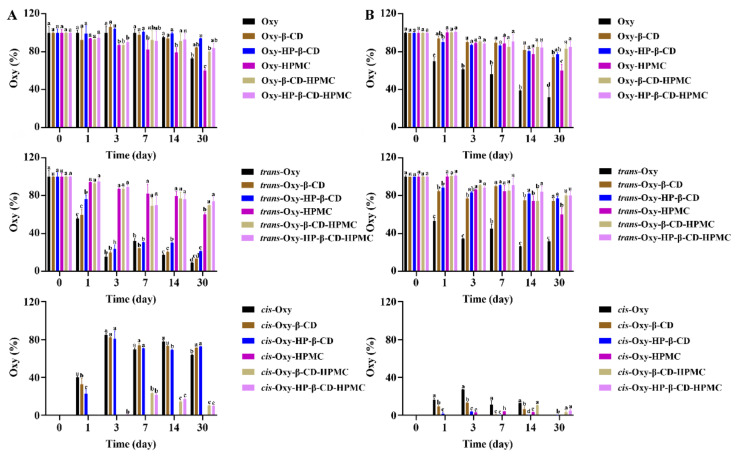
The percentage of remaining Oxy (both *trans*- and *cis*-forms) in Oxy, Oxy–HPMC, Oxy–CD, and Oxy–CD–HPMC solutions during 30 days of storage at 25 °C with light was recorded by monitoring Oxy content with HPLC at a certain time interval (*n* = 3, if no error bars are displayed, errors were smaller than symbols). Data were presented as mean ± SD, and significant differences compared to the pure Oxy group are shown with lower case letters a–d. All samples were stored in well-closed glass bottles. (**A**) Water; (**B**) acidic solution (pH 3.6).

Previous studies have proved that *trans*-form stilbene compounds can be converted into *cis*-structure under light [50]. However, both *trans*- and *cis*-forms are unstable and easily degraded [51]. As shown in Figure 6A top and Table 2, the Oxy–Content in the solution showed a marked reduction, as a consequence of long-term storage. Significant levels of *cis*-Oxy were generated in Oxy solution after storing for 1 day at 25 °C (Figure 6A bottom). The total retention rate of Oxy was 96.19%, 41.92% of which was in the *cis*-form. After 30 days, the retention rate reduced to 72.91%, but the *trans* structure only occupied 9.04% (Figure 6A medium). Compare with pure Oxy, both Oxy–β-CD and HP–β-CD inclusion complexes exhibited an obvious protective effect; however, the proportion of isomerization was hardly decreased. After storage, 13.23% and 21.30% of *trans*-Oxy were detected in Oxy–β-CD and HP–β-CD inclusion compound solutions, respectively, while the retention rates of *cis*-Oxy were 71.48% and 72.64%, respectively. Approximately 60.01% of total Oxy remained after storage in HPMC solution. It is worth noting that almost no *cis*-Oxy was formed in the HPMC solution, which is very consistent with the earlier findings that viscosity is the main solvent property that affects the isomerization kinetics [52]. The Oxy–CD–HPMC ternary complex has the advantages of CD and HPMC. Neither Oxy–β-CD–HPMC nor HP–β-CD–HPMC ternary complex will weaken the stability of Oxy; in contrast, it can improve the retention rate of Oxy to a certain extent. On the other hand, compared with the CD inclusion compound with the absence of HPMC, only a small proportion of isomerization occurred in the ternary complex. At the end of storage, there was no significant difference in the level of *trans*- and *cis*-Oxy in Oxy–β-CD–HPMC and Oxy–HP–β-CD–HPMC solutions, which were both above 70% and 10%, respectively. The above data indicated that forming an inclusion complex with CD can effectively protect Oxy from degradation, and the HPMC in the solution improves the structural stability of Oxy significantly. Together, these data indicate that the poor stability of Oxy in aqueous solution is implicated in the highly susceptible oxidative degradation and isomerization.

**Figure 7:**
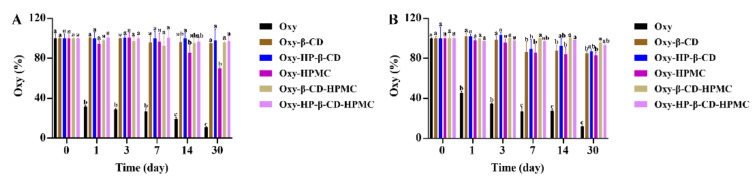
The percentage of remaining Oxy (both *trans*- and *cis*-forms) in Oxy, Oxy–HPMC, Oxy–CD, and Oxy–CD–HPMC solutions during 30 days of storage at 5 °C without light was recorded by monitoring Oxy content with HPLC at a certain time interval (*n* = 3, if no error bars are displayed, errors were smaller than symbols). Data were presented as mean ± SD, and significant differences compared to the pure Oxy group are shown with lower case letters a–d. All samples were stored in well-closed glass bottles. (**A**) Water; (**B**) acidic solution (pH 3.6). The lack of figures of the *trans* and *cis* forms here indicates that no *cis*-Oxy is formed in this treatment, and all remaining Oxy is in the *trans* structure.

**Figure 8:**
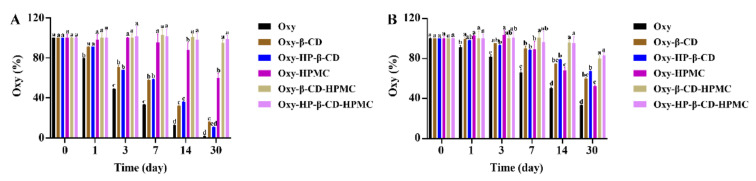
The percentage of remaining Oxy (both *trans*- and *cis*-forms) in Oxy, Oxy–HPMC, Oxy–CD, and Oxy–CD-HPMC solutions during 30 days of storage at 50 °C without light was recorded by monitoring Oxy content with HPLC at a certain time interval (*n* = 3, if no error bars are displayed, errors were smaller than symbols). Data were presented as mean ± SD, and significant differences compared to the pure Oxy group are shown with lower case letters a–d. All samples were stored in well-closed glass bottles. (**A**) Water; (**B**) acidic solution (pH 3.6). The lack of figures of the *trans*- and *cis*-forms here indicates that no *cis*-Oxy is formed in this treatment, and all remaining Oxy is in the *trans* structure.

**Figure 9:**
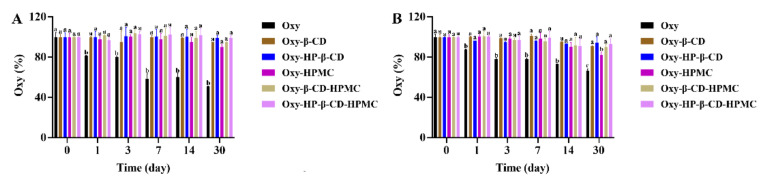
The percentage of remaining Oxy (both *trans*- and *cis*-forms) in Oxy, Oxy–HPMC, Oxy–CD, and Oxy–CD–HPMC solutions during 30 days of storage at 25 °C without light was recorded by monitoring Oxy content with HPLC at a certain time interval (*n* = 3, if no error bars are displayed, errors were smaller than symbols). Data were presented as mean ± SD, and significant differences compared to the pure Oxy group are shown with lower case letters a–d. All samples were stored in well-closed glass bottles. (**A**) Water; (**B**) acidic solution (pH 3.6). The lack of figures of the *trans*- and *cis*-forms here indicates that no *cis*-Oxy is formed in this treatment, and all remaining Oxy is in the *trans* structure.

**Figure 10:**
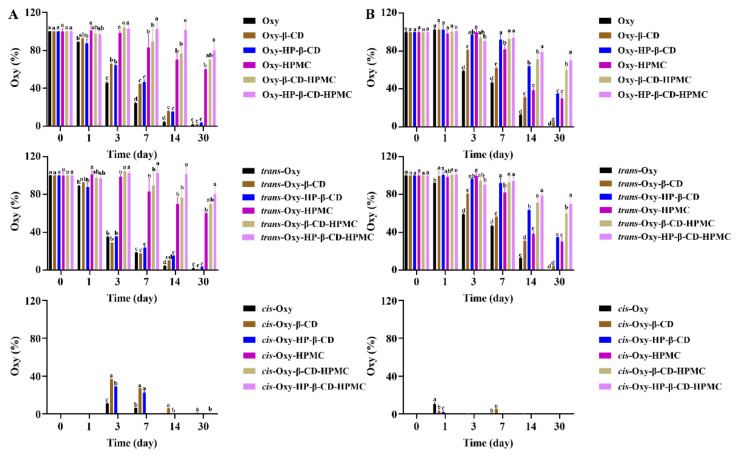
The percentage of remaining Oxy (both *trans*- and *cis*-forms) in Oxy, Oxy–HPMC, Oxy–CD, and Oxy–CD–HPMC solutions during 30 days of storage at 50 °C with light was recorded by monitoring Oxy content with HPLC at a certain time interval (*n* = 3, if no error bars are displayed, errors were smaller than symbols). Data were presented as mean ± SD, and significant differences compared to the pure Oxy group are shown with lower case letters a–e. All samples were stored in well-closed glass bottles. (**A**) Water; (**B**) acidic solution (pH 3.6).

Neither low temperature nor high temperature is suitable for the storage of pure Oxy solution. Refrigeration storage will cause Oxy to precipitate in the solution, while a large amount of Oxy is degraded under the high temperature (Figure 7A and Figure 8A). Light, rather than temperature changes, induces the conversion of *trans*-Oxy molecules to *cis*-form (Figure 8A, Figure 9A and Figure 10A). During storage at 25 °C under light, it is obvious that the isomerization of a *trans*-form towards its *cis*-forms reduces the concentration of the *trans*-form Oxy, which may be related to the reduced activity of Oxy after storage [15,16]. However, compared with the *trans*-form, it is clear that the *cis*-Oxy has better stability in water, thereby the dark condition cannot significantly increase the retention rate of the total Oxy. Forming an inclusion complex can effectively prevent Oxy from being degraded, while the HP–β-CD inclusion complex showed a stronger stabilizing ability than that of the β-CD inclusion compound. The addition of HPMC had a good protective effect on both oxidative degradation and isomerization of Oxy, and the formation of the ternary complex exhibited an excellent stability effect in aqueous solutions, which greatly improved the photothermal stability of Oxy. HPMC in the system may contribute more to this result than CD because it displayed similarly to HPMC, but with better Oxy protection effect. As discovered by Fan et al., HPMC in the solution can prevent oxidation by isolating the small molecules in micelles from oxygen [53]. On the other hand, interaction studies have demonstrated that the binding affinity of Oxy–HP–β-CD was greater than β-CD, while increased temperature and the presence of HPMC will influence this affinity [46]. The corresponding changes in the protective effect of CD on Oxy can be observed in our data. Based on the above findings, it can be inferred that the protection of Oxy by CD in the solution may be related to the binding strength between them. The stronger bonding strength led to a better protective effect.

#### 3.3.3. The Stability of Oxy in Acidic Solution

The study showed that the total Oxy amount in acidic solution exhibits a significantly higher degradation rate than water, accompanied by improved structural stability (Table 2 and Figure 6B). Over 3 days of storage, the retention rate of total Oxy was 61.77%, of which *trans*-forms accounted for 34.56%. The retention rate reduced to 31.94% after 30 days, and all of them were *trans*-Oxy. The stabilizing effect of CD inclusion compounds was also improved compared with water. There was no significant difference between the retention rate of Oxy–β-CD and HP–β-CD inclusion compounds, which were all above 74% at the five detection time points. However, HP–β-CD inclusion complexes exhibited a better protective effect on Oxy than β-CD. The addition of HPMC had a good stabilizing effect on Oxy in solution, where the content of 60.01% Oxy was measured after being placed at 25 °C for 30 days, and almost no isomerization was detected. The Oxy–CD–HPMC solution showed the best storage stability, in which Oxy content remained above 80% of the original after 30 days.

Combined with the results of Figure 7, Figure 8, Figure 9 and Figure 10B, it can be found that temperature changes and light conditions will significantly affect the storage stability of Oxy with the same behavior as in water. The difference is that the Oxy isomerization detected in acidic solutions is much lower under the same storage environment, probably because the *cis* structure is stable near pH neutrality [54]. Compared with neutral solutions, acidic conditions seem to be more suitable for the storage of pure Oxy. This has been previously demonstrated by Zupančič et al., that stilbene compounds are relatively stable in the acidic pH range and are not prone to degradation [55]. The increased protective effect of CD may be related to the stronger bonding strength between the two under an acidic environment. However, the decrease of pH will not further enhance the stability of Oxy in the ternary complex. On the contrary, its protective effect is not as good as water at high temperatures.

### 3.4. Anti-Browning Effects of Oxy–CD–HPMC and Oxy–CD–HPMC–VC on Fresh-Cut Apple Slices

**Figure 11:**
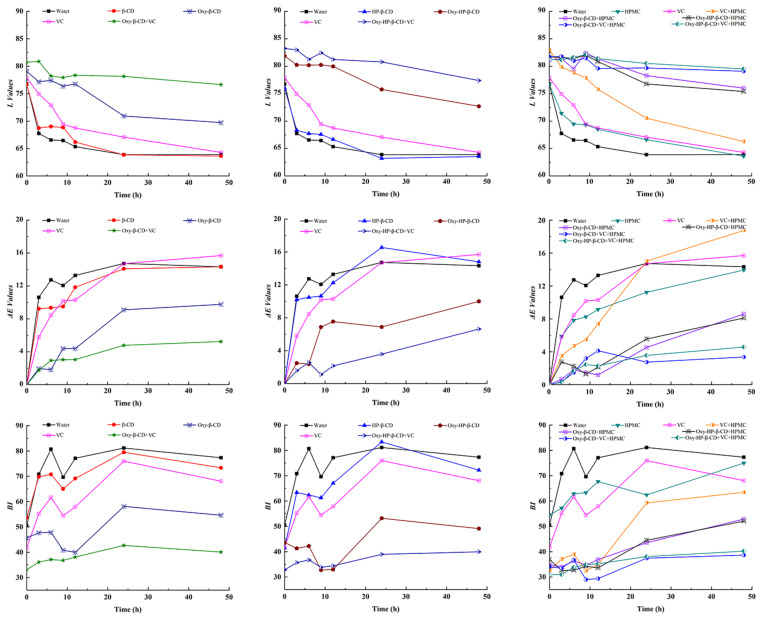
Reflectance measurement of L, Δ*E* values, and *BI* of apple slices soaked in different solutions and stored at room temperature for 48 h.

We have previously demonstrated the anti-browning effect of Oxy–CD on fresh grape juice, indicating that the inclusion of CD does not affect the tyrosinase inhibitory activity of Oxy itself [28]. It has also been proved that Oxy exhibited a good effect on inhibiting the browning of fresh-cut lotus slices [21], and the combination with VC improved the antibrowning effects on some fruits and vegetables greatly [56,57]. Oxy may have a synergistic effect with another antioxidant through a certain mechanism, that is, one antioxidant helps to regenerate the other [10]. In this study, fresh-cut apple slices were used as a model to re-evaluate the characteristics of Oxy–CDs in fresh-cut fruits, as well as the effects of adding HPMC and VC to the solution. The degree of browning of apple slices was monitored by visual evaluation together with the change of L, Δ*E* values, and *BI* [58]. The L value represents brightness, and the larger the L value indicates the smaller the browning, while the Δ*E* value represents the color difference which reflects the color change between two timepoints. When Δ*E* < 1.5, the difference can be considered invisible to the eyes [58]. *BI* represents the purity of brown color and is reported as another important parameter during storage [43]. It can be seen from Figure 11 that as the storage time increases, the apple slices in each experimental group varied degrees of browning, exhibiting reduced L value and a higher Δ*E* value and *BI*.

Water and CD hardly showed the antibrowning effects on apple slices, and HPMC solution showed a weak anti-browning effect, which may be because HPMC forms a thin film on the surface of fruit slices, which acts as an effective semi-permeable barrier to block the contact of fruit slices with oxygen and water vapor in the air to a certain extent, thereby slowing down the rate of browning [59]. The role of similar polysaccharides as edible coatings in improving the quality and shelf life of harvested vegetables has been reported [59,60]. The antibrowning ability of VC alone was also very limited, which disappeared after 3 h. The Oxy–CD inclusion compound (0.5 mg/mL) exhibited excellent ability in maintaining the freshness of apple slices, which is consistent with the findings of Li et al. [56], indicating the potential value of Oxy as an antibrowning agent for fruit slices. As we reported earlier, the co-addiction with VC showed a stronger antibrowning effect than a single compound, indicating that these two components may had a synergistic effect in this process. There was no significant difference between the antibrowning effect of Oxy–β-CD and HP–β-CD inclusion compound. The addition of HPMC does not affect the biological activity of neither VC nor Oxy, in contrast, combining HPMC with VC or Oxy–CD further enhanced their inhibitory effect on browning. Besides its own film-forming effect, another reason may be attributed to the protective effect of HPMC on bioactive components, resulting in the improvement of their antibrowning effect, as the data reported above [21]. The solution containing Oxy–CD, VC, and HPMC showed the best antibrowning effect in all test solutions.

## 4. Conclusions

In this study, Oxy–β-CD, Oxy–HP–β-CD, Oxy–β-CD + HPMC, and Oxy–HP–β-CD + HPMC solutions were prepared and characterized, and their chemical and physical stabilities and antibrowning effects on fresh-cut apple slices were also evaluated. The results demonstrated that adding HPMC to the Oxy–CD inclusion compound solution could effectively improve the stability of Oxy in an aqueous solution, especially in an acidic solution. Similar to the binary system, Oxy–Could still form inclusion compounds with β-CD and HP–β-CD in a ratio of 1:1 in HPMC solution (1 mg/mL), and the presence of HPMC helped to further improve the binding affinity of CD and Oxy. Interaction studies showed that HPMC could interact with Oxy–CD inclusion compounds and increase the binding affinity of Oxy and CD. It is speculated that when three of them exist together in the solution, Oxy may enter the cavity of CD, and HPMC will be wrapped outside the inclusion compound. The antibrowning results of the Oxy–β-CD, Oxy–HP–β-CD, Oxy–β-CD + HPMC, and Oxy–HP–β-CD + HPMC solutions suggested that the formation of inclusion complexes with β-CD and HP–β-CD would not affect the antibrowning ability of Oxy. However, when Oxy–CD inclusion complexes were used combined with VC and HPMC, it showed excellent antibrowning effects for apple slices. Taken together, it is suggested that Oxy–β-CD/Oxy–HP–β-CD + HPMC solutions have great potential as antibrowning agents for the food industry in terms of enhanced efficacy and stability, and they could be employed as antibrowning agents to extend the shelf-life of fresh-cut fruit slices. This discovery provides a new way to improve fruit quality and shelf life by incorporating anti-browning compounds into edible coatings.

## Figures and Tables

**Table 1:** Each experimental group solution for processing apple slices.

Test Solution	Composition
Water	Water
β-CD	0.4 mg/mL β-CD
HP–β-CD	0.4 mg/mL HP–β-CD
HPMC	0.1 mg/mL HPMC
VC	0.5 mg/mL VC
Oxy–β-CD	0.5 mg/mL Oxy–β-CD inclusion complex
Oxy–HP–β-CD	0.5 mg/mL Oxy–HP–β-CD inclusion complex
Oxy–β-CD + VC	0.5 mg/mL Oxy–β-CD inclusion complex and 0.5 mg/mL VC
Oxy–HP–β-CD + VC	0.5 mg/mL Oxy–HP–β-CD inclusion complex and 0.5 mg/mL VC
VC + HPMC	0.5 mg/mL VC and 0.1 mg/mL HPMC
Oxy–β-CD + HPMC	0.5 mg/mL Oxy–β-CD inclusion complex and 0.1 mg/mL HPMC
Oxy–HP–β-CD + HPMC	0.5 mg/mL Oxy–HP–β-CD inclusion complex and 0.1 mg/mL HPMC
Oxy–β-CD + VC + HPMC	0.5 mg/mL Oxy–β-CD inclusion complex, 0.5 mg/mL VC and 0.1 mg/mL HPMC
Oxy–HP–β-CD + VC + HPMC	0.5 mg/mL Oxy–HP–β-CD inclusion complex, 0.5 mg/mL VC and 0.1 mg/mL HPMC

**Table 2:** The percentage of remaining Oxy (total Oxy, *trans*- and *cis*-forms) in Oxy, Oxy–HPMC, Oxy–CD, and Oxy–CD–HPMC solutions during 30 days of storage at 25 °C with light.

Water							
	Time (Day)	Oxy	Oxy–β-CD	Oxy–HP–β-CD	Oxy–HPMC	Oxy–β-CD-HPMC	Oxy–HP–β-CD-HPMC
Total Oxy	0	100 ± 6.50 a	100 ± 0.69 a	100 ± 4.03 a	100 ± 3.48 a	100 ± 0.86 a	100 ± 0.54 a
	1	96.19 ± 2.85 a	92.60 ± 12.36 a	99.04 ± 8.49 a	94.39 ± 0.29 a	93.02 ± 0.90 a	94.93 ± 3.29 a
	3	100.07 ± 4.05 a	106.24 ± 2.69 a	104.39 ± 0.82 a	87.28 ± 0.74 b	87.37 ± 1.28 b	90.22 ± 2.10 b
	7	101.81 ± 4.19 a	98.01 ± 3.02 a	101.21 ± 0.81 a	82.33 ± 9.89 b	92.53 ± 10.14 ab	91.71 ± 10.11 ab
	14	95.20 ± 1.31 a	93.97 ± 1.36 a	99.50 ± 1.38 a	79.51 ± 4.90 b	91.25 ± 8.00 a	93.15 ± 6.47 a
	30	72.91 ± 1.04 a	84.71 ± 0.28 a	93.93 ± 0.65 b	60.01 ± 2.00 a b	80.22 ± 0.87 a	84.12 ± 4.72 ab
*Trans*-Oxy	0	100 ± 6.50 a	100 ± 0.69 a	100 ± 4.03 a	100 ± 3.48 a	100 ± 0.86 a	100 ± 0.54 a
	1	54.27 ± 1.57 c	59.87 ± 6.06 c	76.28 ± 4.37 b	94.39 ± 0.29 a	93.02 ± 0.90 a	94.93 ± 3.29 a
	3	15.22 ± 2.09 b	19.81 ± 1.86 b	23.44 ± 4.26 b	87.28 ± 0.74 a	87.37 ± 1.28 a	89.14 ± 1.99 a
	7	32.02 ± 2.66 b	24.23 ± 0.70 b	30.55 ± 0.40 b	82.33 ± 9.89 a	69.01 ± 8.24 a	69.98 ± 8.89 a
	14	17.35 ± 0.32 c	20.71 ± 0.59 c	30.21 ± 0.65 b	79.51 ± 4.90 a	77.05 ± 6.73 a	76.04 ± 5.21 a
	30	9.04 ± 0.17d	13.23 ± 0.18 cd	21.30 ± 0.48 c	60.01 ± 2.00 b	70.01 ± 0.24 a	74.01 ± 4.65 a
*Cis*-Oxy	0	ND	ND	ND	ND	ND	ND
	1	41.92 ± 1.28 a	32.73 ± 6.30 b	22.76 ± 4.13 c	ND	ND	ND
	3	84.85 ± 1.96 a	82.44 ± 0.83 a	80.95 ± 8.05 a	ND	ND	1.08 ± 0.11 b
	7	69.79 ± 1.52 a	73.78 ± 2.32 a	70.66 ± 0.41 a	ND	23.52 ± 1.9 b	21.73 ± 1.23 b
	14	77.85 ± 1.00 a	73.26 ± 0.76 a	69.28 ± 0.72 b	ND	14.21 ± 1.28 c	17.11 ± 1.26 c
	30	63.87 ± 0.87 b	71.48 ± 0.10 a	72.64 ± 0.17 a	ND	10.21 ± 0.63 c	10.11 ± 0.07 c
Acidic solution (pH 3.6)							
	Time (day)	Oxy	Oxy–β-CD	Oxy–HP–β-CD	Oxy–HPMC	Oxy–β-CD-HPMC	Oxy–HP–β-CD-HPMC
Total Oxy	0	100 ± 0.95 a	100 ± 0.02 a	100 ± 0.06 a	100 ± 2.37 a	100 ± 0.21 a	100 ± 0.88 a
	1	70.08 ± 3.69 c	93.83 ± 2.67 ab	90.16 ± 3.03 b	100.30 ± 2.29 a	100.72 ± 0.66 a	101.04 ± 2.13 a
	3	61.77 ± 0.80 b	90.34 ± 1.29 a	87.31 ± 1.06 a	89.28 ± 0.84 a	91.05 ± 1.24 a	88.67 ± 1.28 a
	7	56.42 ± 9.07 b	89.52 ± 2.26 a	86.61 ± 1.04 a	88.69 ± 6.28 a	85.24 ± 6.79 a	90.94 ± 7.66 a
	14	39.25 ± 0.55 b	81.88 ± 4.60 a	80.87 ± 1.19 a	77.61 ± 5.73 a	85.12 ± 4.81 a	84.39 ± 5.68 a
	30	31.94 ± 9.95 d	74.59 ± 2.08 b	77.33 ± 1.87 ab	60.01 ± 7.03 c	83.22 ± 4.43 a	85.12 ± 5.98 a
*Trans*-Oxy	0	100 ± 0.95 a	100 ± 0.02 a	100 ± 0.06 a	100 ± 2.37 a	100 ± 0.21 a	100 ± 0.88 a
	1	53.57 ± 2.00 c	84.54 ± 1.29 b	88.37 ± 1.47 b	100.3 ± 2.29 a	100.72 ± 0.66 a	101.04 ± 2.13 a
	3	34.56 ± 0.54 c	77.1 ± 0.95 b	83.43 ± 0.81 ab	86.27 ± 0.79 a	91.05 ± 1.24 a	88.67 ± 1.28 a
	7	45.33 ± 4.59 b	90.11 ± 1.12 a	91.12 ± 0.61 a	84.64 ± 6.04 a	85.24 ± 6.79 a	90.94 ± 7.66 a
	14	26.43 ± 0.44 c	75.37 ± 2.25 b	82.16 ± 0.76 a	74.3 ± 5.40 b	74.43 ± 12.52 b	84.39 ± 5.68 a
	30	31.94 ± 0.85 c	74.59 ± 2.08 a	77.33 ± 1.87 a	60.01 ± 7.03 b	80.01 ± 2.73 a	80.01 ± 4.5 a
*Cis*-Oxy	0	ND	ND	ND	ND	ND	ND
	1	16.51 ± 1.7 a	9.28 ± 1.37 b	1.79 ± 1.56 c	ND	ND	ND
	3	27.21 ± 0.26 a	13.25 ± 0.34 b	3.88 ± 0.25 c	3.01 ± 0.04 c	ND	ND
	7	11.09 ± 4.48 a	0 ± 1.14 c	0 ± 0.43 c	4.05 ± 0.24 b	ND	ND
	14	12.83 ± 0.11 a	6.51 ± 2.35 b	0 ± 0.43 d	3.31 ± 0.33 c	10.7 ± 0.35 a	ND
	30	ND	ND	0 ± 0.17 b	ND	3.21 ± 1.70 a	5.11 ± 1.48 a

Data were presented as mean ± SD, and significant differences compared to the pure Oxy group are shown with lower case letters a–d. “ND” represents not detected. All samples were stored in well-closed glass bottles.

## Data Availability

The data presented in this study are available on request from the corresponding author.

## Supplementary Materials

The following supporting information can be downloaded at: https://www.mdpi.com/article/10.3390/foods11162471/s1, Figure S1: High-Performance Liquid Chromatography (HPLC) analysis of *trans*- and *cis*-Oxy; Figure S2: Standard curves for *trans*- and *cis*-Oxy; Figure S3: Ultra Performance Liquid Chromatography-Mass Spectrometry (UPLC-MS).
