# Supplementary material for: Characterization, Stability, and Antibrowning Effects of Oxyresveratrol Cyclodextrin Complexes Combined Use of Hydroxypropyl Methylcellulose
Source: Foods. 2022 Aug 16;11(16):2471. doi: 10.3390/foods11162471 (PMC9407340; doi:10.3390/foods11162471)
Supplement: Supplementary file 1 [file foods-11-02471-s001.zip › foods-1851316-supplementary.pdf]

foods

Supplementary Materials

Characterization, Stability, and Antibrowning Effects of Oxyresveratrol Cyclodextrin Complexes Combined Use of Hydroxypropyl Methylcellulose

Jianfei He [1,2], Huai-Yu Chen [1], Hongbin Chen [1], Baobei Wang [1], Fengxian Guo [1] and Zong-Ping Zheng [1,*]

[1] Fujian Province Key Laboratory for the Development of Bioactive Material from Marine Alge, College of Oceanology and Food Science, Quanzhou Normal University, Quanzhou 362000, China
[2] Department of Biomedical Science, University of Copenhagen, Copenhagen, Denmark
* Correspondence: zzpsea@qztc.edu.cn; Tel.: +86 595 22919563

High-Performance Liquid Chromatography (HPLC) analysis

The concentration of *trans*-/*cis*-Oxy was determined by Waters 2707 autosampler with 2487 dual-wavelength and absorbance detector set at 325 nm, using a reverse-phase GraceSmart (4.6 μm, 2.1×250 nm, Ryss Tech Ltd., China); 0.1% formic acid (phase A) and methanol (phase B) were used as mobile phase. The analysis conditions were as follows: 0 min, 20% B; 0-15 min, 20%-45% B; 15-20 min, 45%-60% B; 20-23 min, 60%-100% B; 23- 25 min, 100% B; 25-27 min, 100%-20% B; 27-30 min, 20% B. Flow rate was set at 1.0 mL/min, and the injection volume was 10 μL. Three injections were performed for each sample. Retention times: *cis*-Oxy, 11.2 min; *trans*-Oxy, 13.4 min.

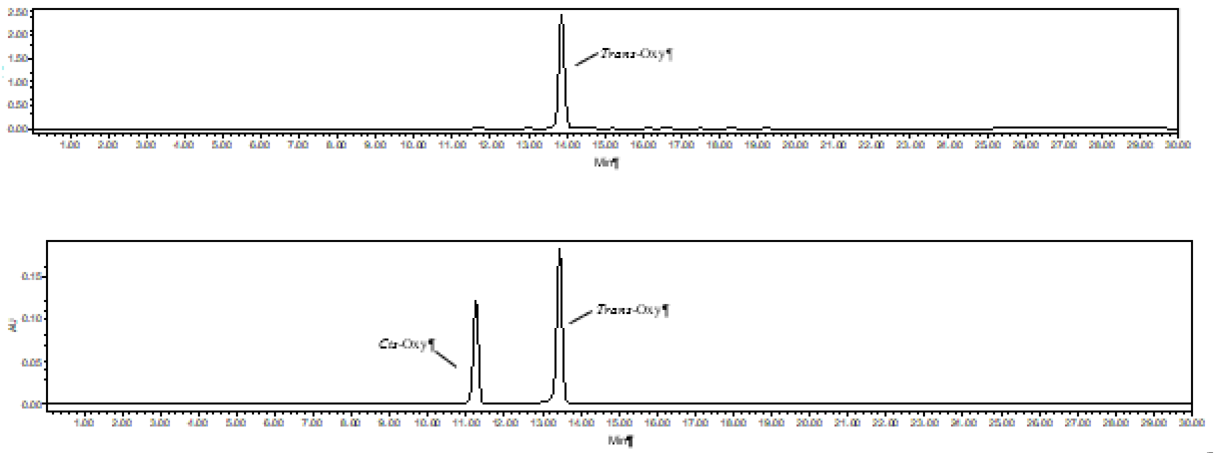

Figure S1. High-Performance Liquid Chromatography (HPLC) analysis.

Standard curves for *trans*- and *cis*-Oxy

Values obtained for methanolic *trans*-Oxy showed linearity over the concentration range of 3.125-200 μg/mL and was linear with a correlation coefficient of 0.9999. *Cis*-Oxy was obtained by irradiating a 20 mL stock methanol solution of *trans*-Oxy (200 μg/mL) for certain time, while *trans*-Oxy was UV-irradiated through a clear glass sealed bottle vial placed under a three-purpose ultraviolet analyzer WFH-203(ZF-1) at 365 nm. During the illumination process, samples (100 μL each) were taken at time points of 30 min, 1, 2, 3, and 5 h, and HPLC was performed immediately after sampling. Three injections were performed for each sample. Values obtained for methanolic *cis*-Oxy showed linearity over the concentration range of 9.37-175.48 μg/mL and was linear with a correlation coefficient of 0.9995.

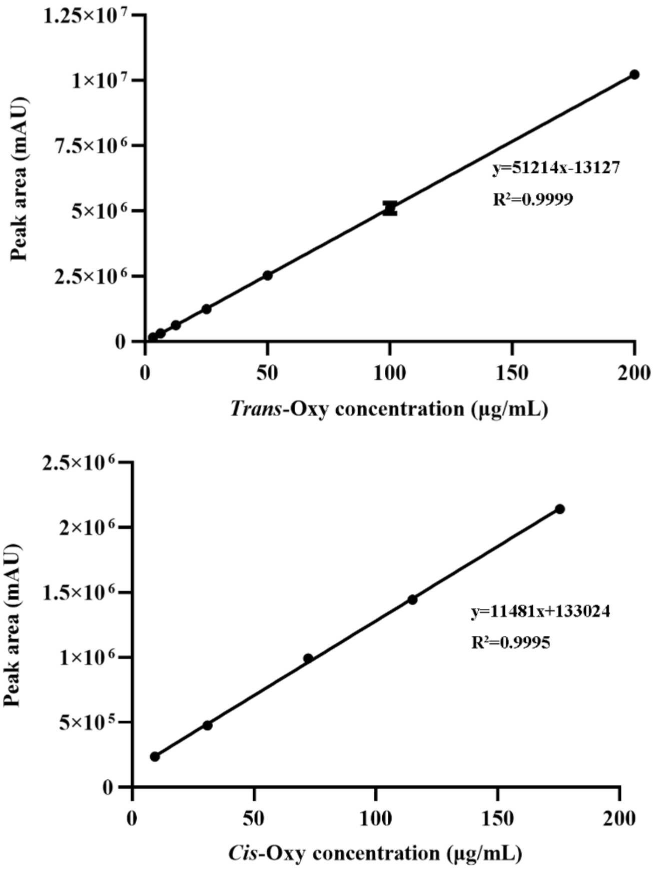

Figure S2. Standard curves for *trans-* and *cis-*Oxy.

Ultra-Performance Liquid Chromatography Mass Spectrometry (UPLC-MS)

The UPLC-MS analysis was performed on a Waters Acquity UPLC equipped with a Waters Acquity photodiode array detector (PAD) and a Waters Maldi Synapt quadrupole time-of-flight (Q-TOF) mass spectrometer (MS; Waters Corporation, Milford, Massachusetts, USA) equipped with an ESI ionization source in the negative/positive mode. Separation of *trans-*/*cis-*Oxy compounds was achieved using a BEH C-18 packed column (1.7 μm, 2.1×100 mm) (Milford). The column temperature was 35 °C; 0.1% formic acid (phase A) and acetonitrile (phase B) were used as mobile phase. The analysis conditions for LC were as follows: 0.1 min 10% B; 0.1-8 min 70% B; 8-12 min 100% B, return to initial condition for 0.1 min. Flow rate was set at 0.3 mL/min, and the injection volume was 5 μL. The mass spectra were obtained by electrospray ionization in negative-ion mode at an optimized condition as follows: capillary and sampling cone voltage were 3.0 kV and 20 V, respectively; source temperature was 100 °C; desolvation temperature was 400 °C; cone and desolvation gas flow (nitrogen) were 50 L/h and 700 L/h, respectively.

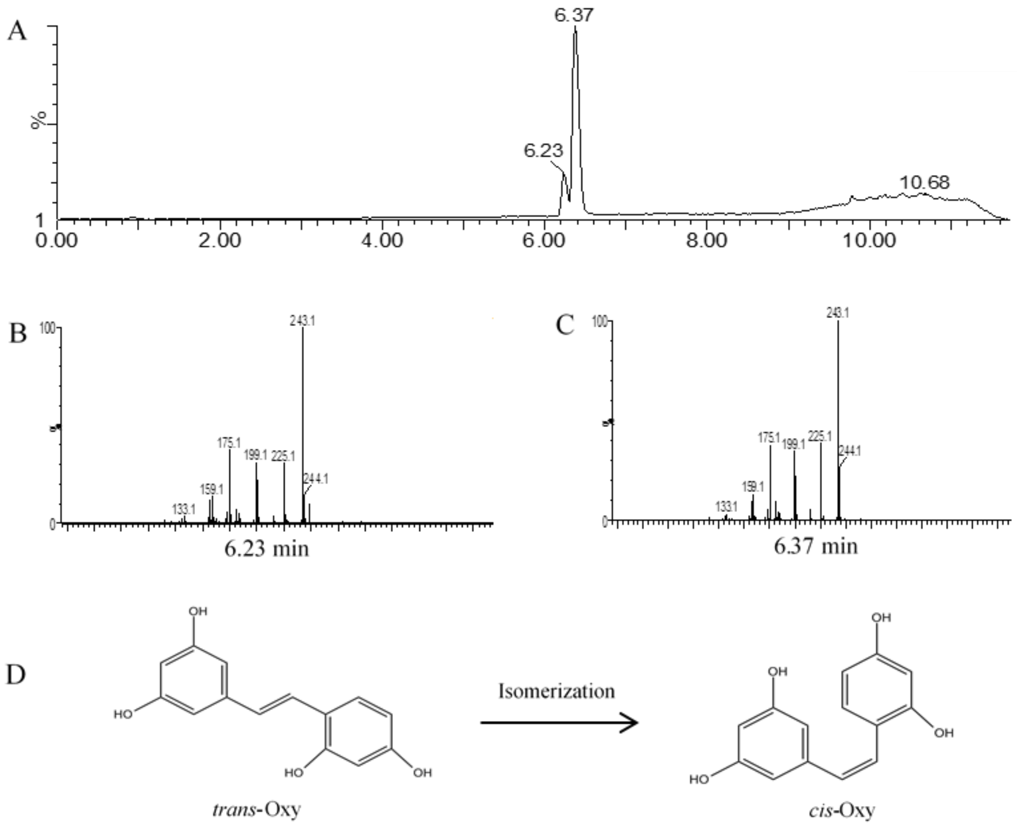

Figure S3. Ultra-Performance Liquid Chromatography-Mass Spectrometry (UPLC-MS).
